# The effects of alerting signals in masked priming

**DOI:** 10.3389/fpsyg.2013.00448

**Published:** 2013-07-17

**Authors:** Rico Fischer, Franziska Plessow, Andrea Kiesel

**Affiliations:** ^1^Department of Psychology, Technische Universität DresdenDresden, Germany; ^2^Department of Psychology, Julius-Maximilians-Universität WürzburgWürzburg, Germany

**Keywords:** temporal predictability, alerting signal, accessory, masked priming, action-trigger, target primes, novel primes

## Abstract

Alerting signals often serve to reduce temporal uncertainty by predicting the time of stimulus onset. The resulting response time benefits have often been explained by facilitated translation of stimulus codes into response codes on the basis of established stimulus-response (S-R) links. In paradigms of masked S-R priming alerting signals also modulate response activation processes triggered by subliminally presented prime stimuli. In the present study we tested whether facilitation of visuo-motor translation processes due to alerting signals critically depends on established S-R links. Alerting signals resulted in significantly enhanced masked priming effects for masked prime stimuli that included and that did not include established S-R links (i.e., target vs. novel primes). Yet, the alerting-priming interaction was more pronounced for target than for novel primes. These results suggest that effects of alerting signals on masked priming are especially evident when S-R links between prime and target exist. At the same time, an alerting-priming interaction also for novel primes suggests that alerting signals also facilitate stimulus-response translation processes when masked prime stimuli provide action-trigger conditions in terms of programmed S-R links.

## The effects of alerting signals in masked priming

Task-irrelevant acoustic signals that precede an imperative visual target stimulus by few hundred milliseconds (e.g., 200–1000 ms) have been demonstrated to improve performance, typically reflected in speeded responses (Niemi and Näätänen, 1981). The presence of such an alerting signal can be utilized as readiness signal predicting the temporal onset of the forthcoming stimulus and thus reducing temporal uncertainty by attentional focusing. At the same time, alerting signals also elicit a brief surge of arousal that non-specifically primes low-level motor pathways (Sanders, 1983).

Accordingly, much research has demonstrated that beneficial effects of alerting signals occur on various levels of information processing, including perceptual encoding and sensory information accumulation (Bausenhart et al., 2010; Seibold et al., 2011), early response selection processes (Hackley and Valle-Inclán, 1999) and/or motor execution processes (Miller et al., 1999; Kiesel and Miller, 2007; Thomaschke and Dreisbach, 2013).

On a more general level, the functional role of alerting signals may be to support the cognitive system in adapting behavior to an expected event by increasing unspecific alertness and motor readiness and by inducing a bias toward stronger reliance on reflex-like habitual behavior (Fischer et al., 2013). This assumption is captured in the recently proposed facilitated response activation account of alerting signals, suggesting that alerting signals facilitate automatic translation of stimulus codes into response codes (Fischer and Plessow, in revision; Fischer et al., 2010, 2012). In particular, it is argued that alerting signals lead to a more efficient transmission of perceptual information of the expected stimulus into corresponding motor codes. In line with assumptions of increased information transmission efficiency, recent findings show that the presence of alerting signals reduce neural activity in the primary visual cortex (Fischer et al., 2013). More specifically, alerting signal effects of facilitated behavioral responses correlated with a reduction in the neural activity in the primary visual cortex. Thus, expectation of a sensory input reduces the neural effort needed to process this visual stimulus (Alink et al., 2010). Therefore, information transmission from lower to higher cortices is achieved with less neural activation (Rao and Ballard, 1999), which is in line with an assumed beneficial alerting signal based visuo-motor translation.

This facilitated visuo-motor translation by alerting signals might be based on direct (i.e., learned) S-R links that are established by responding to a stimulus and thus actively associating a particular stimulus or stimulus feature with the corresponding motor response (Neumann and Klotz, 1994; Klapp and Haas, 2005). Currently there is some evidence that alerting signals impact on these types of S-R links (see below), whereas clear impact of alerting signals on visuo-motor translation without direct S-R links is to date lacking. Evidence for facilitated response activation on the basis of direct S-R links can be found in often reported alerting-congruence interactions when alerting signals are incorporated in conflict paradigms (e.g., Simon, Eriksen flanker).^1^ In such paradigms conflict occurs when relevant and irrelevant information activate different response alternatives (incongruent trials) compared to the activation of the same response alternative (congruent trials). Consequently, response conflicts, for example, reflect competition between simultaneously activated response codes. In this context, the presence of alerting signals is assumed to facilitate automatic stimulus-response translation processes for relevant and for irrelevant stimulus attributes, resulting in increased interference effects between simultaneously active response codes (e.g., Fischer et al., 2010; Böckler et al., 2011). In a recent electrophysiological study, for example, Böckler et al. (2011) found that an alerting-signal increased the amplitude of the lateralized readiness potential (LRP) for the incorrect response in incongruent trials, which has been taken as direct evidence that alerting signals facilitate visuo-motor response activation.

Importantly, in a previous behavioral study we demonstrated that facilitation of visuo-motor translation due to alerting signals was only observed when direct stimulus-response links existed. In a word-variant of the Eriksen flanker task (Shaffer and LaBerge, 1979; Fischer and Schubert, 2008) increased interference due to alerting signals was found only for flanker items that were included in the response set and thus contained direct stimulus-response associations. Distracter words that were not part of the response set revealed semantic conflict that was, however, not affected by alerting signals (Fischer et al., 2012).

The beneficial effects of alerting signals on visuo-motor translation processes can also be found for response activation processes triggered by subliminally presented (masked) stimuli (Fischer et al., 2007). For example, in a masked priming paradigm (Vorberg et al., 2003), participants were asked to respond to left or right pointing arrows. Unbeknownst to the participants, target arrows were preceded by masked prime arrows that also pointed toward the left or right side and thus formed congruent or incongruent prime-target relations when pointing into the same or the opposite direction than the target arrow, respectively. Alerting signals were presented in various random (Experiment 1) or blocked (Experiment 2) foreperiod intervals prior to the prime-target pair. Importantly, alerting signals facilitated visuo-motor response activation processes triggered by the visual stimuli. As a consequence enlarged masked priming effects were especially observed when alerting signals preceded the target arrow by at least 250 ms compared to conditions with shorter foreperiods or conditions without alerting signals.

Importantly, prime arrows were able to subconsciously activate stimulus-response links. Alerting signals served to increase this prime-triggered response activation. More specifically, alerting signals facilitated transmission of information along the established stimulus-response links. Because recent data suggested that in conflict tasks increased effects due to alerting signals depend on existing stimulus-response links (Fischer et al., 2012), in the present study we aimed to extend these findings by further testing and specifying the stimulus-response link dependency.

In a masked number comparison task, for example, in which participants categorize target digits for example as smaller or larger than five (Dehaene et al., 1998; Naccache and Dehaene, 2001; Kunde et al., 2003; Reynvoet et al., 2005; Kiesel et al., 2006a, 2007b; Van den Bussche et al., 2009; Fischer et al., 2011) two sets of prime stimuli can be included. First, primes that also appear as target stimuli are referred to as “target primes” (e.g., the digits 1, 4, 6, and 9). Stimulus-response links are established whenever a target number is responded to with a specified response key (e.g., digits larger than five—right response). These response activation processes on the basis of stimulus-response links are triggered when the same target stimuli serve as masked primes in other trials (Neumann and Klotz, 1994; Damian, 2001). Second, prime stimuli that never serve as target stimuli and are therefore never responded to are called “novel primes”. Importantly for the aim of the present study, these novel primes do not contain established direct stimulus-response links. In fact, some researches assume that novel primes elicit semantic processing (Naccache and Dehaene, 2001; Reynvoet et al., 2005; see Van den Bussche et al., 2009 for an overview). The differential reliance on established direct stimulus-response links for target and novel primes may account for observed differences in processing triggered by these prime types. For example, masked priming by novel primes has been shown to be smaller in size (Naccache and Dehaene, 2001), to depend on task conditions (Kiesel et al., 2006a; Pohl et al., 2010; Fischer et al., 2011), and has been reported to display a different time course (Kinoshita and Hunt, 2008; Finkbeiner and Friedman, 2011).

In the present study we aim to extend and further test the assumption that the presence of alerting signals affect visuo-motor translation particularly on the basis of established S-R links and not on the basis of semantic processing (Fischer et al., 2012). For this, we implemented a different paradigm than in Fischer et al. (2012), i.e., masked priming paradigm including target and novel prime stimuli that are known to differ with respect to the involvement of established direct stimulus-response links. If alerting signals exclusively facilitate visuo-motor response activation on the basis of established direct S-R links, alerting signals should increase masked priming effects specifically for target but not for novel primes.

## Experiment 1

The aim of Experiment 1 was to test whether alerting signals affect response activation processes triggered by target primes that include S-R links (see also Fischer et al., 2007) and response activation processes triggered by novel primes. For this, we included an alerting signal (present vs. absent) in a masked number priming task (Naccache and Dehaene, 2001), in which the numbers 1, 4, 6, and 9 served as target and as target primes, whereas the enclosed numbers 2, 3, 6, and 7 functioned as novel primes.

### Method

#### Participants

Thirty-two students of the Technische Universität Dresden (24 female, 21–35 years; mean age ± SD, 25.0 ± 2.8 years) participated in the study for partial course fulfillment or €5 payment. All participants had normal or corrected-to normal vision and were naive about the hypothesis of the experiment.

#### Apparatus and stimuli

Stimulus presentation and collection of responses were performed by an IBM-compatible computer with a 17 inch VGA-Display. Participants responded by pressing the “X” and “,” key of a standard QWERTZ keyboard with the left and right index finger, respectively. Stimulus presentation and data recording were realized using Presentation software (Version 0.71, Neurobehavioral Systems). Stimulus presentation was synchronized with the vertical retraces of a 70-Hz monitor, resulting in a vertical refresh rate of approximately 14 ms. Two sets of stimuli were used that were presented white on black background. The numbers 1, 4, 6, and 9 served as prime and as target stimuli (target primes) whereas the numbers 2, 3, 7, and 8 where never presented as targets and thus, served as prime stimuli only (novel primes). Out of a set of fourteen masks, each consisting of randomly assigned capitalized/non-capitalized 7 letter strings chosen from the whole alphabet (e.g., TsPLqaF), one was randomly selected to serve as pre-mask. From the same set another mask was randomly selected to serve as post-mask. With a viewing distance of about 60 cm, the visual angle extended to 0.38° × 0.76° for prime and target stimuli and to 3.34° × 0.76° for masks. A tone of 700 Hz frequency served as alerting signal and was presented binaurally via headphones.

#### Procedure

Participants were asked to perform a size judgment task (smaller or larger than 5) on numbers between 1 and 9, excluding 5, responding with the left index finger to numbers smaller than five and with the right index finger to numbers larger than five. A masked prime stimulus preceded the target number. They described a congruent relation when both numbers fell on the same side of five. In an incongruent condition, prime and targets resided on opposite sides of five. In order to prevent prime visibility, a prime stimulus was imbedded between two masks, each consisting of a random letter string, e.g., WLulMBa (see Dehaene et al., 1998).

Trials without an alerting signal started with the presentation of a fixation cross for 1100 ms, which was followed by a pre-mask for 71 ms. Subsequently, a prime stimulus was shown for 43 ms and was immediately masked by a post-mask for 57 ms. Finally a target number was presented for 200 ms. If a response exceeded 1800 ms (beginning at target onset) or if the wrong response was given, the feedback “too slow” or “error” was presented for 300 ms. A correct response was followed by the fixation cross for another 300 ms. Following feedback, the fixation sign was presented in a random response-stimulus-interval (RSI) that varied in steps of 100 ms in the range between 1100 and 2000 ms. In half of the trials an alerting signal was presented 250 ms prior to the pre-mask. Instructions emphasized speed and accuracy of responding to equal parts.

The experiment consisted of 768 trials presented in 12 blocks separated by brief pauses. Each block comprised 64 trials corresponding to a combination of Novel or Target prime (4 + 4) × Target (4) × Alerting signal (2). The experiment was preceded by 16 practice trials.

After the priming experiment participants were fully informed about the presence of the prime stimuli. We conducted a signal detection experiment in which participants were asked to discriminate whether a prime was smaller or larger than five. Participants were instructed to respond at leisure and to prioritize accuracy over speed. To avoid the possibility of unconscious priming influencing the free response choice (Schlaghecken and Eimer, 2004; Kiesel et al., 2006a,b), we included an interval of 1000 ms after target onset, in which in case of an executed response the feedback “too fast” was provided (adopted from Vorberg et al., 2003).

### Results

#### Prime visibility

To assess prime visibility, we computed the signal detection measure *d*′ whereby primes smaller than 5 were treated as signal. Overall discrimination for primes was *d*′ = 1.70 and deviated from zero, *t*_(31)_ = 13.20, *p* < 0.001. Discrimination performance was better for novel than for target primes, *t*_(31)_ = 8.73, *p* < 0.001, it amounted to *d*′ = 2.26 for novel primes and *d*′ = 1.26 for target primes. Due to the high prime visibility, we further investigated whether target and novel priming effects were related to prime visibility. To pursue this aim we conducted a regression analysis as proposed by Draine and Greenwald (1998; see also Greenwald et al., 1995). We calculated a priming index for each participant and prime-type: prime index = 100 × (RT incongruent—RT congruent)/RT congruent. Individual target and novel priming indices were regressed onto the individual d′ values for target and novel primes, respectively. No correlation between d′ and the corresponding target priming effects, *r* = − 0.153, *p* = 0.403, or novel priming effects, *r* = 0.217, *p* = 0.234, were found. Similarly, none of the correlations were significant (all *p*'s > 0.147) when considering target and novel prime indices separately for alerting signal present vs. alerting signal absent. These findings show that despite the high visibility values, the size of target and novel priming effects seemed not to depend on prime visibility.

#### Priming task

**Figure 1:**
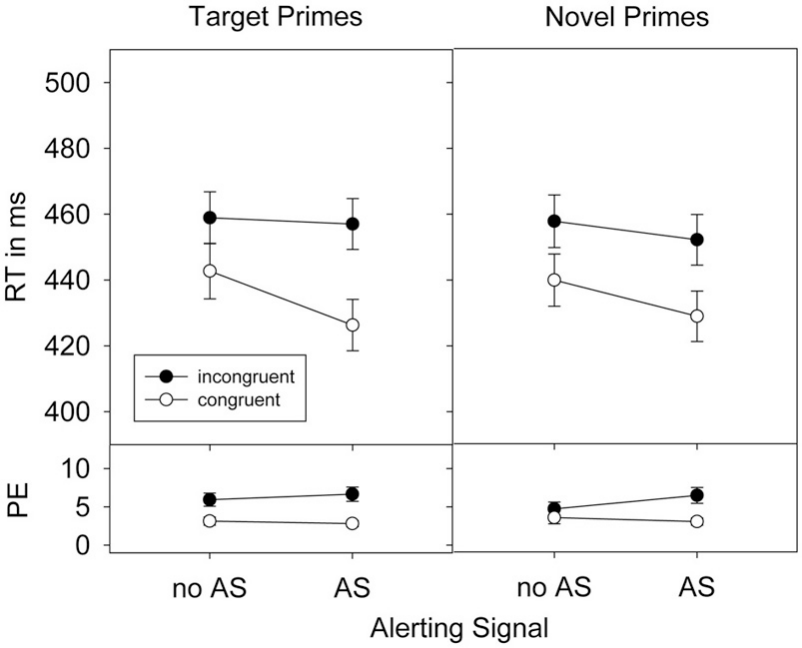
**Response times (RTs), standard errors of the means, and percent error (PE) in Experiment 1 as a function of prime-target congruence, prime type, and alerting signal (AS)**.

For the RT analyses, all error trials and trials following an error were discarded (8.7%). Furthermore, all trials that did not fit the outlier criterion (RTs <150 and > 1200 ms) were also excluded from analyses (0.1%). Prior to the error analysis, only trials following an error were eliminated. Repeated measures ANOVAs were conducted on mean RTs and percent error containing the factors Alerting signal (present, absent), Congruence (C vs. IC) and Prime-type (target vs. novel primes). Results are presented in Figure 1.

#### RT

Responses were faster when an alerting signal was present (441 ms) than when it was absent (450 ms), *F*_(1, 31)_ = 32.09, *p* < 0.001, η^2^_*p*_ = 0.51. Prime stimuli shortened RTs in congruent prime-target relations (434 ms) compared to incongruent prime-target relations (456 ms), *F*_(1, 31)_ = 115.76, *p* < 0.001, η^2^_*p*_ = 0.79. This priming effect was not differentially affected by the factor prime-type, *F*_(1, 31)_ = 1.54, *p* = 0.224, η^2^_*p*_ = 0.05. However, the priming effect was increased by the presence of an alerting signal, *F*_(1, 31)_ = 23.93, *p* < 0.001, η^2^_*p*_ = 0.44. This increase was stronger for target primes than for novel primes as indicated in the significant 3-way interaction between Alerting signal, Congruence, and Prime-type on RTs, *F*_(1, 31)_ = 5.23, *p* < 0.05, η^2^_*p*_ = 0.14.

**Figure 2:**
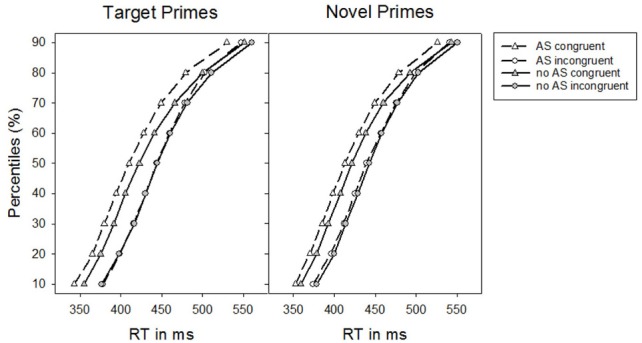
**Percentiles of participants' response times (RTs) in Experiment 1 as a function of the absence vs. presence of an alerting signal for target primes and novel primes, respectively**.

We conducted RT distribution analyses (De Jong et al., 1994; Kinoshita and Hunt, 2008; Fischer et al., 2010) to test whether alerting signals impact on different time segments of the RT distribution for target and novel primes, respectively. For this, we computed the percentile values based on the whole RT distribution. That is, we assessed the upper border for each percentile and therewith the 50% percentile is the median. The distribution analysis showed that the specific alerting signal impact on priming effects for target and novel primes did not differ across different RT bins, as the three-way interaction between Alerting signal, Congruence, and Prime-type was not further modulated by the factor Percentile (10, 20, 30, 40, 50, 60, 70, 80, and 90), *F* < 1. Priming effects generally decreased as a function of increasing RTs, *F*_(8, 248)_ = 11.32, *p* < 0.001, η^2^_*p*_ = 0.27 [*F*_(1, 31)_ = 13.52, *p* = 0.001, η^2^_*p*_ = 0.30, linear contrast], which, however, was the same for target and novel primes, *F* < 1. The impact of the alerting signal on the overall masked priming effect was also independent of the time course, *F* < 1 (see Figure 2).

Separate ANOVAs for each prime-type confirmed an alerting signal based increase of the priming effect for target primes, *F*_(1, 31)_ = 20.48, *p* < 0.001, η^2^_*p*_ = 0.40. In particular, a priming effect of 16 ms, *t*_(31)_ = 6.09, *p* < 0.001, in conditions without an alerting signal increased to a priming effect of 31 ms, *t*_(31)_ = 9.00, *p* < 0.001, when an alerting signal was present. For novel primes, however, the priming effect in conditions without an alerting signal [18 ms, *t*_(31)_ = 7.46, *p* < 0.001] also increased significantly when an alerting signal was present [23 ms, *t*_(31)_ = 9.61, *p* < 0.001], *F*_(1, 31)_ = 4.74, *p* < 0.05, η^2^_*p*_ = 0.13.

When effects of repetition priming were controlled for (i.e., elimination of exact prime-target stimulus repetitions), target primes revealed a response priming effect that was significantly increased by the presence of an alerting signal,*F*_(1, 31)_ = 12.66, *p* < 0.01, η^2^_*p*_ = 0.29. Although this increase of response priming was numerically still larger than the analogous alerting signal based increase for priming by novel primes, this difference was only marginally significant, *F*_(1, 31)_ = 3.46, *p* = 0.072, η^2^_*p*_ = 0.10.

#### Errors

A total of 4.5% errors were observed in Experiment 1. The alerting signal did not affect overall error rates, *F* < 1, ruling out the possibility of a speed-accuracy trade-off. Error rates were modulated by prime congruence, *F*_(1, 31)_ = 21.90, *p* < 0.001, η^2^_*p*_ = 0.41. More errors were committed in incongruent (5.9%) than in congruent (3.1%) prime-target relations. This priming effect was more pronounced for target (3.3%) than for novel primes (2.3%), *F*_(1, 31)_ = 6.39, *p* < 0.05, η^2^_*p*_ = 0.17, and in conditions with (3.6%) compared to conditions without (2.0%) an alerting signal, *F*_(1, 31)_ = 6.12, *p* < 0.05, η^2^_*p*_ = 0.17. A significant three-way interaction, however, was not observed, *F*_(1, 31)_ = 1.14, *p* = 0.294, η^2^_*p*_ = 0.04.

### Discussion

In Experiment 1 the presence of an alerting signal resulted in increased masked priming effects. This holds especially for masked priming elicited by target prime stimuli which also serve as to-be-categorized target stimuli thus containing overtly established S-R links. Importantly, masked priming effects induced by novel primes that were never overtly responded to, were also affected by the presence of alerting signals. This finding in particular demonstrates that alerting signals can affect response activation processes triggered by stimuli that do not include direct S-R links (for a further discussion, see the General Discussion section).

At the same time, the alerting signal based increase of masked priming for target primes was larger in size than the increase of masked priming found for novel primes. Restricting the analysis exclusively to trials of stimulus-response priming (i.e., excluding identical prime-target pairs), the stronger influence of alerting signals on priming by target primes compared to novel primes was still detectable but fell short of significance. Importantly, the influence of the alerting signal on the masked priming effect was the same across the RT distribution for target and novel primes. As in Kinoshita and Hunt (2008), priming effects for target and novel primes declined with increasing RT bins. In contrast to Kinoshita and Hunt (2008), however, both functions for target and novel primes declined in the same way.

## Experiment 2

Experiment 2 served to replicate findings from Experiment 1 and therefore, to provide further evidence for an alerting signal based increase of masked priming effects for target as well as for novel primes. Two changes were included. First, because of rather high prime detection rates in Experiment 1, the prime stimulus duration was shortened. Second, we increased the variation in RSI to reduce an overall temporal predictability of trial onset.

### Methods

#### Participants

Twenty-six new students of the Technische Universität Dresden (17 female, 18–33 years; mean age ± SD, 21.9 ± 3.5 years) participated in the study for partial course fulfillment or €5 payment. All participants had normal or corrected-to normal vision and were naive about the hypothesis of the experiment.

#### Apparatus, stimuli and procedure

The experimental setup of Experiment 2 varied to that in Experiment 1 as follows: Stimuli were presented on a 17 inch VGA-Display with the vertical retraces of a 75-Hz monitor. This resulted in a vertical refresh rate of approximately 13.3 ms. The pre-mask was presented for 67 ms and the subsequent prime stimulus was shown for two refresh cycles of the display (27 ms). The prime was followed by a brief blank (13 ms) and a post-mask shown for 53 ms. In addition, the variation in the range of RSIs was extended. Experiment 2 included ten RSIs increasing from 300 to 2100 ms in steps of 300 ms. The RSI was selected randomly in each trial.

### Results

#### Prime visibility

Overall discrimination for primes was *d*′ = 0.64 and deviated from zero, *t*_(25)_ = 5.74, *p* < 0.001. Discrimination performance was again better for novel (*d*′ = 0.78) than for target primes (*d*′ = 0.51), *t*_(25)_ = 2.49, *p* < 0.05. The regression analyses, however, revealed no correlation between *d*′ and the corresponding target priming effects, *r* = 0.172, *p* = 0.400, and the corresponding novel priming effects, *r* = − 0.109, *p* = 0.596. As in Experiment 1, none of the correlations were significant (all *p*'s > 0.107) when considering target and novel prime indices separately for alerting signal present vs. alerting signal absent.

#### Priming task

**Figure 3:**
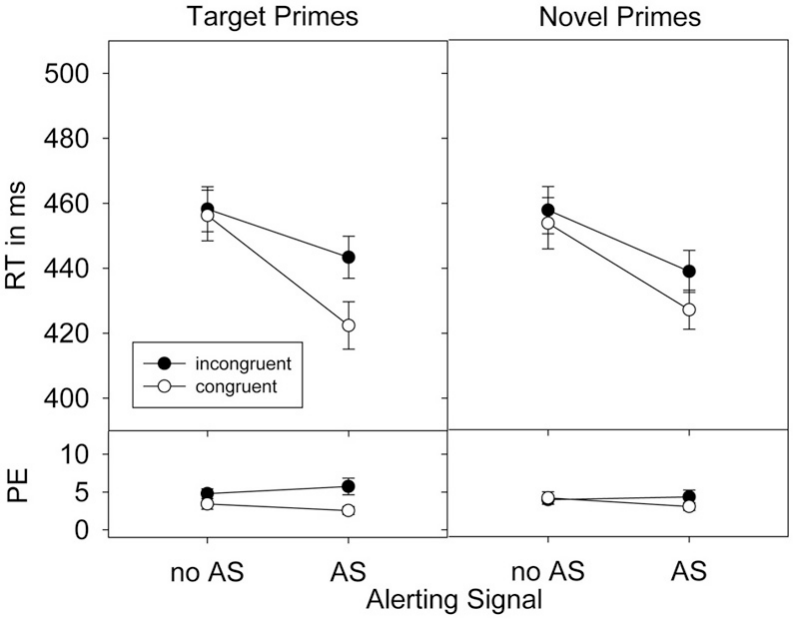
**Response times (RTs), standard errors of the means, and percent error (PE) in Experiment 2 as a function of prime-target congruence, prime type, and alerting signal (AS)**.

As in Experiment 1, all error trials and trials following an error were discarded (7.6%) and all trials that did not fit the outlier criterion (RTs <150 ms and > 1200 ms) were also excluded from analyses (<0.1%). Prior to the error analysis, only trials following an error were eliminated. Repeated measures ANOVAs were conducted on mean RTs and percentage error containing the factors Alerting signal (present, absent), Congruence (C vs. IC) and Prime-type (target vs. novel primes). Results are presented in Figure 3.

#### RT

The presence (433 ms) compared to the absence (456 ms) of an alerting signal considerably reduced RTs, *F*_(1, 25)_ = 103.43, *p* < 0.001, η^2^_*p*_ = 0.81. The factor Congruence also affected responses, *F*_(1, 25)_ = 16.33, *p* < 0.001, η^2^_*p*_ = 0.40, with faster responses in congruent (440 ms) than in incongruent (449 ms) prime-target relations. This priming effect was increased by an alerting signal, *F*_(1, 25)_ = 21.84, *p* < 0.001, η^2^_*p*_ = 0.47. As in Experiment 1, this alerting signal based increase of the priming effect was larger for target compared to novel primes, as indicated by the significant three-way interaction of all factors, *F*_(1, 25)_ = 5.70, *p* < 0.05, η^2^_*p*_ = 0.19 (see also Figure 3). Finally, the priming effect in general seemed larger for target than for novel primes, which however, failed significance, *F*_(1, 25)_ = 3.01, *p* = 0.095, η^2^_*p*_ = 0.11.

**Figure 4:**
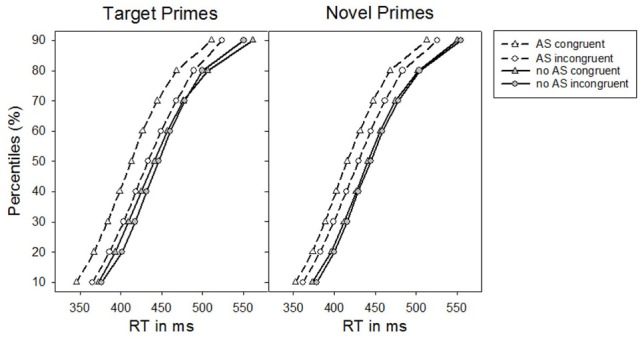
**Percentiles of participants' response times (RTs) in Experiment 2 as a function of the absence vs. presence of an alerting signal for target primes and novel primes, respectively**.

Similar to Experiment 1, the RT distribution analysis showed that there was no interaction between the factors Alerting signal, Congruence, Prime-type, and Percentile, *F*_(8, 200)_ = 1.00, *p* = 0.392, η^2^_*p*_ = 0.04. Yet, irrespective of prime-type, the impact of the alerting signal on priming seemed less pronounced for the slowest RTs of the RT distribution, *F*_(8, 200)_ = 3.38, *p* = 0.026, η^2^_*p*_ = 0.12 [*F*_(1, 25)_ = 6.54, *p* = 0.017, η^2^_*p*_ = 0.21, linear contrast]. Finally, although masked priming effects for novel primes were rather stable across the RT distribution, masked priming effects for target primes declined at larger percentiles resulting in an interaction between Congruence, Prime-type, and Percentile (see Figure 4), *F*_(8, 200)_ = 4.27, *p* = 0.021, η^2^_*p*_ = 0.15 [*F*_(1, 25)_ = 5.20, *p* = 0.031, η^2^_*p*_ = 0.17, linear contrast].

Separate ANOVAs for target and novel primes confirmed an increase of the priming effect by an alerting signal for target primes, *F*_(1, 25)_ = 24.06, *p* < 0.001, η^2^_*p*_ = 0.49, as well as for novel primes, *F*_(1, 25)_ = 4.82, *p* < 0.05, η^2^_*p*_ = 0.16. For target primes the presence of an alerting signal increased from a non-significant priming effect of 2 ms, *t*_(25)_ = 0.86, *p* = 0.397, to a significant 21 ms, *t*_(25)_ = 4.58, *p* < 0.001 priming effect. Similar results were obtained for novel primes. Here, a non-significant effect of 4 ms, *t*_(25)_ = 1.71, *p* = 0.100, without alerting signal was increased to 12 ms, *t*_(25)_ = 3.85, *p* < 0.01 when an alerting signal was present.

As in Experiment 1, the elimination of prime-target stimulus repetitions for target primes resulted in a priming effect that increased when an alerting signal was presented, *F*_(1, 25)_ = 19.53, *p* < 0.001, η^2^_*p*_ = 0.44. The alerting signal based increase in priming was still larger for target primes than for novel primes, *F*_(1, 25)_ = 4.23, *p* < 0.05, η^2^_*p*_ = 0.15.

#### Errors

Participants committed a total of 4.0% errors. As in Experiment 1, the alerting signal did not affect overall error rates, *F* < 1, ruling out the possibility of a speed-accuracy trade-off. However, more errors were produced in incongruent than in congruent prime-target relations, *F*_(1, 31)_ = 8.02, *p* < 0.01, η^2^_*p*_ = 0.24. Furthermore, this priming effect was larger for target (2.3%) than for novel primes (0.5%), *F*_(1, 31)_ = 10.25, *p* < 0.01, η^2^_*p*_ = 0.29 and also when an alerting signal was present (2.2%) than when it was absent (0.6%), *F*_(1, 31)_ = 5.96, *p* < 0.05, η^2^_*p*_ = 0.19. Again, a significant three-way interaction, however, was not observed, *F* < 1.

### Discussion

Experiment 2 closely replicated findings from Experiment 1 providing virtually the same results and therefore, making a strong case of alerting signals affecting not only masked priming by target primes but also increasing masked priming revealed by novel primes. In addition, the alerting signal based increase of masked priming effect for target primes also exceeded the increase for novel primes when the analysis was restricted to stimulus-response priming (excluding identical prime-target pairs). As in Experiment 1, the influence of the alerting signal on the masked priming effect was the same across the RT distribution for target and novel primes. Although, the impact of alerting signals on priming effects was stronger for faster RTs, this finding did not depend on prime-type. Again, this suggests that the alerting signal impact on priming effects for target and novel primes relates to the same RT bins. At the same time, in Experiment 2 and in contrast to Experiment 1 we found a stronger decline of priming effects for target than for novel primes.

## General discussion

In two experiments it was tested whether alerting signals affect response activation processes in a masked priming paradigm with two different types of prime stimuli that differed with respect to the involvement of learned direct S-R links. In the implemented number categorization task target primes consisted of numbers that also served as target stimuli. By overtly responding to these stimuli, S-R links are established on the basis of which response activation processes are triggered when these stimuli serve as masked primes. Novel primes consisted of a set of numbers that were never presented as target stimuli. Participants did not overtly execute a smaller or larger than five response to these stimuli so that no overt S-R links are formed.

According to previous studies, in which it was assumed that alerting signals particularly facilitate visuo-motor translation processes on the basis of established S-R links (Fischer et al., 2010, 2012), it was argued that the presence of alerting signals (compared to the absence of alerting signals) increase the priming effect especially for target primes for which S-R links existed. In support with this assumption, in two experiments an enhanced masked priming effect for target primes was consistently demonstrated under alerting signal stimulation. An open question was whether alerting signals also affect masked priming for novel primes that did not include direct S-R links. Results of both experiments showed that response activation processes triggered by novel primes were also affected by the presence of alerting signals, resulting in increased masked priming effects for novel primes. Importantly, even when restricting masked priming by target primes to pure stimulus-response priming, the effects of alerting signals on the size of the masked priming effects was more pronounced for target primes than for novel primes. At the same time, even though detectability of prime stimuli (*d*′) was not zero, neither the masked priming effect seemed to depend on prime visibility nor was prime visibility different for target and novel primes.

Together these results have important implications. First, alerting signals seem to especially facilitate response activation processes that are triggered by visual stimuli when established S-R links exist (Fischer et al., 2012) as in the case for target primes. In addition, smaller but reliable effects of alerting signals on the size of masked priming effects for novel primes suggest that the effects of alerting signals seem not exclusively depend on overtly established S-R links. Furthermore, alerting signals affect priming by target and novel primes similarly across different RT bins.

How do these findings fit with previous studies demonstrating that alerting signals facilitate response activation processes when S-R links exist, but do not facilitate semantic processing in conditions without S-R links (Fischer et al., 2012)?

One possible explanation is based on the action-trigger account (Kunde et al., 2003), which does not posit semantic processing for novel primes. Instead, the extent to which novel primes trigger response activation processes that result in priming effects depends on whether these prime stimuli belong to the action-trigger set. According to this account, stimuli trigger responses when they match existing action release conditions, so called action triggers that automatically activate the related action (cf. Kiesel et al., 2007a). In particular, following the instruction participants form memory representations of environmental events that are thought to activate specific motor responses (i.e., action triggers). Online processing, however, is characterized by a comparison process that defines whether a given stimulus matches the established action triggers. If so, the related response alternative is automatically activated.

For example, in the applied number priming task of the present study, the digits 1 and 4 might serve as action triggers for the left response (smaller than five) and the digits 6 and 9 might serve as action triggers for the right response (larger than five). The overt categorization of target stimuli according to the task rule results in an inclusion of unseen prime stimuli into the set of action triggers (cf. Kiesel et al., 2007a, 2009). Moreover, and in line with common assumptions of a mental left-to-right spatial representation of numbers (i.e., mental number line, Galton, 1880; Göbel et al., 2001; Fias and Fischer, 2005), action triggers established for numbers 1, 4, 6, and 9 may also extend to mentally enclosed numbers of novel primes, i.e., 2, 3, 7, and 8, thus explaining priming effects revealed by novel primes without an assumed semantic processing (Kunde et al., 2003). In order to test the assertion of the action-trigger account, Kunde and colleagues varied the set of target and novel primes. For example, using numbers adjacent to five (i.e., 3, 4, 6, and 7) as target stimuli resulted in priming effects when the same stimuli served as target primes. At the same time, however, neighboring but not enclosed novel primes (i.e., 1, 2, 8, and 9) did not yield a priming effect (Kunde et al., 2003, Experiment 2).

Back to our own study, alerting signals seem to facilitate performance whenever stimuli are able to trigger automatic response activation processes. That is, novel primes that are included in the action trigger set automatically trigger response activation processes that can be modulated by the presence of alerting signals. This alerting signal based modulation of response activation occurs at the same RT bins for target primes and for novel primes. Therefore, it is conceivable that in the present study, and in contrast to Fischer et al. (2012), participants were able to form very specific action-trigger (S-R links) because the expected stimuli were clear defined. That is, similarly to Kunde et al. (2003), numbers representing novel primes were included into the action-trigger set and were able to automatically trigger response activation processes.

Therefore, the present findings of alerting signals modulating masked priming effects by novel primes also suggest that processing of novel primes is not (exclusively) based on semantic processing (but see Van den Bussche et al., 2009). Although we cannot exclude that additional components of (e.g., semantic) processing may kick in for novel primes especially at larger RT bins (Kinoshita and Hunt, 2008), alerting signals affected target and novel prime processing irrespective of RT bins (Experiment 1) and across the same RT bins (Experiment 2). Furthermore, the fact that we did not find unequivocal evidence for differential time courses for target and novel priming effects, clearly calls for further research in this line. Instead, we think that novel primes that are included in the action-trigger set form so-called programmed or instructed S-R links which are formed for expected stimuli as soon as participants read and implement the task instruction (Woodworth, 1938; Hommel, 2000). In line with the action-trigger account, alerting signals not only affect response activation processes of overtly learned and responded to S-R links, but also affect response activation processes for those stimuli that do not contain direct learned S-R links but which are part of the action trigger condition set.

On a more broadly applied and more speculative note, given that alerting signals are often implemented as trigger signals to facilitate the activation of motor responses in dangerous situations (e.g., facilitating the initiation of an emergency stop when driving a car), extending the impact of alerting signals from highly practiced visuo-motor links also to less practiced but instructed visuo-motor links seem encouraging news. More specifically, it may be useful to also apply alerting signals as trigger signals to facilitate the activation of instructed but less practiced, often only theoretical motor programs (e.g., to counter steer or to full braking).

### Conflict of interest statement

The authors declare that the research was conducted in the absence of any commercial or financial relationships that could be construed as a potential conflict of interest.

## Footnotes

^1^At present it is debated whether alerting-congruence interactions can also be found in Stroop paradigms, in which relevant and irrelevant information are included into a single object representation (Fischer and Plessow, in revision; Weinbach and Henik, 2012).
